# Current Status of Antimicrobial Stewardship Programs in São Paulo Hospitals

**DOI:** 10.6061/clinics/2021/e2882

**Published:** 2021-06-23

**Authors:** Silvia Akemi Sato, Denise Assis Brandão, Geraldine Madalosso, Anna S. Levin, Lauro Vieira Perdigão, Maura Salaroli Oliveira

**Affiliations:** I Departamento de Controle de Infeccao, Hospital das Clinicas HCFMUSP, Faculdade de Medicina, Universidade de Sao Paulo, Sao Paulo, SP, BR; II Divisao de Infeccoes Hospitalares, Centro de Vigilancia Epidemiologica ”Prof. Alexandre Vranjac” Centro de Controle de Doencas, Secretaria de Estado da Saude, Sao Paulo, SP, BR; III Departamento de Doencas Infecciosas, Laboratorio de Investigacao Medica (LIM 54) e Instituto de Medicina Tropical, Universidade de Sao Paulo, Sao Paulo, SP, BR

**Keywords:** Antimicrobial Stewardship, Antibiotics, Antimicrobial Resistance, Statewide Survey

## Abstract

**OBJECTIVES:**

Antimicrobial stewardship programs (ASPs) comprise coordinated interventions designed to improve antimicrobial use. Understanding the current structure of ASP hospitals will support interventions for the improvement of these programs. This study aimed to describe the status of ASPs in hospitals in São Paulo, Brazil.

**METHODS:**

A cross-sectional survey was conducted on the ASPs of hospitals in the state of São Paulo from March to July 2018. Through interviews by telephone or e-mail, we queried which components of the Infectious Diseases Society of America/Society for Healthcare Epidemiology of America and Centers for Disease Control and Prevention guidelines were implemented.

**RESULTS:**

The response rate was 30% (28/93 hospitals), and 26 hospitals (85%) reported having a formal ASP. The most frequently implemented strategies were antimicrobial surgical prophylaxis guidelines (100%), empiric sepsis guidelines (93%), and the presence of ASP team members during bedside rounds (96%). The least commonly implemented strategies included prior authorization for all antimicrobials (11%), pharmacokinetic monitoring, and an adjustment program for patients on IV aminoglycosides (3%). Regarding the metrics of the ASP, the most common indicator was the rate of antimicrobial resistance (77%). Eighteen hospitals evaluated antimicrobial consumption using defined daily dose, and only 29% evaluated the days of therapy; 61% of hospitals reported their results to the hospital administration and 39% to the prescribers.

**CONCLUSIONS:**

Most hospitals have a formal and active ASP, but with timely actions. We observed inconsistencies between what program leaders understand as the main objective of ASP and the metrics used to evaluate it. Part of the effort for the next few years should be to improve program evaluation metrics and to provide feedback to physicians and hospital leadership.

## INTRODUCTION

Antimicrobial stewardship programs (ASPs) have been defined as coordinated interventions designed to optimize the use of antimicrobials (1). These interventions are important because of the several possible benefits to patients in reducing costs and controlling hospital microbial ecology (2). Implementing ASP together with other policies to reduce overuse of antibiotics could save up to 1.6 million lives by 2050 and US$ 4.8 billion per year, according to the Organization for Economic Co-operation and Development (World Health Organization, 2015)**.** One of the most discussed benefits of implementing an ASP is its impact on antimicrobial resistance. The direct consequences of infections by resistant microorganisms can be serious, including increased mortality and length of hospital stay (3).

ASP strategies are variable, and different levels of hospitals have various tools and practices (1). Despite the differences among methods, the implementation depends on the organizational, structural, and cultural context. It is important to emphasize that, despite government efforts toward ASP implementation, many aspects are not well known, such as knowledge of ASP hospitals, actions already implemented, the practitioners' thoughts and beliefs, and the assessment of critical needs and priorities for targeting available resources (4). The objective of this study was to describe the status of ASPs in Brazilian acute care hospitals.

## METHODS

### Setting, participants, and study design

São Paulo State is a Brazilian state with approximately 800 hospitals, of which 425 have adult intensive care units (ICUs). Since 2004, there is a statewide surveillance system to monitor healthcare-associated infections, antimicrobial resistance, and antimicrobial consumption coordinated by São Paulo State Health Department (Center for Epidemiologic Surveillance). We invited 93 hospitals included in a previous study and considered representative of the state (5).

We then conducted a cross-sectional survey to evaluate the ASPs in these hospitals. Recruitment phone calls were made between March and July 2018. After accepting to participate, a survey that addressed points related to ASPs was answered by the participant over the phone or by using electronic form. We requested that the person in charge of the ASP in the organization responded to the survey. Follow-up calls were made to encourage responses. There were no financial incentives to participate in this study.

The survey was designed based on the Infectious Diseases Society of America/Society for Healthcare Epidemiology of America (IDSA/SHEA) and Centers for Disease Control and Prevention (CDC) guidelines (1) that state evidence-based recommendations for implementing an effective ASP.

### Data analysis

Categorical variables were described using frequencies and proportions, and continuous variables were described according to their parametric distributions.

### Ethical approval

This study was approved by the Institutional Committee Ethics in Research (number 032597183.0000.0068). Consent was obtained electronically.

## RESULTS

The survey response rate was 30% (28/93). Half of the respondents were from public hospitals, 25% worked in private hospitals, and 25% worked in philanthropic hospitals. The average number of total ICU beds was 341 and 47, respectively. Among the responders, 85% (26/28) reported having a formal and written ASP. Most ASPs (85%) were led by an infectious disease (ID) physician. Most responders were physicians (71%, 20/28), followed by nurses (21%, 6/28), and 7% (2/28) were pharmacists. All ASPs had physicians as part of their teams. Thirty-two percent of hospitals (9/28) had the support of a nurse, and in 57% (16/28), a pharmacist was part of the ASP staff. Regarding human resources of infection control service, there was an average of 1-5 nurses/200 beds and 1-3 physicians/200 beds.

The strategies, practices, and policies of the surveyed ASP are listed in Table 1. The most common intervention used to optimize antimicrobial usage was a guideline for antimicrobial prophylaxis in surgery (100%) and an ASP team member during bedside rounds (93%); 71% had electronic prescriptions.

## DISCUSSION

Our study provided a cross-sectional view of implemented ASP strategies, practices, and policies at São Paulo hospitals. We revealed that the majority of participating hospitals had formal, written, and active ASPs. Because Brazil has a federal law (issued in 1998) stating that Infection Control Committees are mandatory for all hospitals and that an ASP is part of their duties. We found that most of the implemented optimization strategies were recommended by the IDSA/SHEA guidelines (1). Regarding strategies to improve antimicrobial prescription, we noticed that all facilities had surgical prophylaxis guidelines, and most had sepsis guidelines. Written protocols are undoubtedly an important strategy; however, we consider them the initial step for implementing ASPs. We noticed that in most institutions, a member of the ASP was part of a multidisciplinary round. This strategy allows audit, education, and face-to-face interactions; however, it is time-consuming, and the optimal frequency of participation at these rounds to positively impact antimicrobial prescriptions has not been established (6).

As expected, few hospitals use preauthorization for all antimicrobials, while half of them use a list of selected antimicrobials that require preauthorization for use. Pre-authorization is time-consuming and adds an extra step to the prescription process, resulting in an unacceptable delay in antimicrobial administration. Another disadvantage of pre-authorization is the need for a full-time ID physician consultation. However, it usually provides stricter control of antimicrobial usage (7).

We observed that physicians consider the decrease in antimicrobial resistance as the most relevant impact of implementing ASPs. Most of them use resistance profiles and/or multiresistant infection rates as indicators of ASPs. These rates are known to be influenced by other variables, such as the admission of patients already colonized or infected by multidrug-resistant pathogens, cross-transmission rates, and the existence of effective infection prevention policies (2). Thus, by monitoring these rates alone, it is difficult to attribute either the success or failure of the current strategies of ASPs. In addition to these disadvantages, the positive impact of ASPs in decreasing antimicrobial resistance in health facilities is not well established yet. A recent systematic review found that there are few studies, most of them with inadequate design, and concluded that, to date, “there is no solid evidence that ASPs are effective in reducing antibiotic resistance in hospital settings” (8).

The use of antimicrobial consumption as an outcome of an ASP is frequent. The greatest disadvantage of using this outcome alone is that antimicrobial consumption does not provide information on how antibiotics are used and is greatly influenced by the case-mix. To measure the adequacy of prescriptions, some institutions measure the proportion of adherence to recommendations, however, it is very labor-intensive (9). Although few respondents consider costs as the main priority of ASPs, one-third monitor expenses with antimicrobials. In other words, our perception was that the selected objectives of ASPs do not match the indicators that are monitored and publicized. One of the main findings of this study was the observation of this inconsistency between what program leaders understand as the main objective of ASP and the metrics used to evaluate this inconsistency. In this sense, our results provide some insight into ASP outcomes and metrics. To be manageable and effective, ASPs must have consistent and meaningful metrics with established goals and a surveillance system to monitor and to report them systematically. Finally, we could not find any common measure to all hospitals, making a comparison of performance impossible.

Another important issue to address is the finding that few hospitals can systematically disclose their ASP results to prescribers and leadership. We believe that the absence of systematic reporting is problematic. It is necessary to modify the behavior of prescribing physicians to improve the use of antimicrobials, and it might be easier to achieve this by showing and arguing with real, local, and updated data. This lack of disclosure has already been reported in a similar survey conducted at 101 hospitals in the United States. In this study, the authors found that most hospitals (85%) reported to a committee rather than individuals (10).

The human resources involved in an ASP are crucial for effectiveness. It has been suggested that “ID physicians are uniquely trained, experienced, and best suited to lead ASPs” (11). In this sense, most surveyed facilities have an ID physician as the leader. Unfortunately, it was not possible to evaluate the amount of time each professional dedicated to an ASP. Unlike other studies (10), the presence of pharmacists as co-leaders was not frequent. A microbiology laboratory is crucial for ASP success, and we believe that most hospitals have available automated systems for microorganism identification and *Clostridioides difficile* testing.

Of note, one-third of hospitals do not have electronic prescriptions. It is evident that the use of electronic systems for prescribing enables data capture more quickly and effectively increases patient safety by decreasing errors in prescribing and facilitates the implementation of ASPs (12).

Our study had some limitations. First, a response rate of less than 50% may introduce bias and affect generalizability. Hospitals with no ASPs might choose not to participate. Second, it was not possible to ensure the accuracy of responses. Finally, the statistical analyses were descriptive.

In conclusion, most participating hospitals had a formal and written ASP with an ID physician as the leader. We observed inconsistency between what program leaders understand as the main objective of ASP and the metrics used to evaluate it. Part of the effort for the next few years should be to improve program evaluation metrics and to provide feedback to physicians and hospital leadership.

## AUTHOR CONTRIBUTIONS

Sato SA made substantial contributions to conception, study design, data collection and analysis, and approval of the final version of the manuscript. Brandão DA, Madalosso G and Levin AS contributed to the study design and approved the final version of the manuscript. Perdigão Neto LV contributed to the study design, manuscript drafting and approval of its final version. Oliveira MS made substantial contributions to the conception, study design, data collection and interpretation, manuscript drafting of the manuscript and final approval.

## Figures and Tables

**Table 1:** Description of the antimicrobial stewardship strategies, practices, and policies of 28 hospitals of the state of São Paulo, Brazil.

Strategy	N (%)
**Tools to improve antimicrobial prescription**	
Guideline for antimicrobial prophylaxis in surgery	28 (100)
ID physician available for remote consultation	27 (96)
Presence of ASP team member during bedside rounds	26 (93)
Post prescription auditing	25 (89)
Guideline for empirical prescription in sepsis	24 (86)
Policy requiring prescribers to document indication for all antibiotics	24 (86)
Feedback on prescribing practices	22 (84)
Prescriber education with formal classes	21 (75)
Guideline for antimicrobial use in renal impairment	21 (75)
Systematic evaluation of redundant spectrum	20 (71)
Pharmacokinetic monitoring and adjustment program for patients on IV vancomycin	18 (67)
Determination of vancomycin serum level	17 (61)
Promotion of Intravenous-to-oral antibiotic transition	13 (46)
Prior authorization requirement for selected antimicrobials	13 (46)
Guideline for antimicrobial use in hepatic impairment	11 (39)
Determination of aminoglycoside serum levels	5 (18)
Prior authorization requirement for all antimicrobials	3 (11)
Pharmacokinetic monitoring and adjustment protocol for aminoglycosides	1 (3)
**Availability of microbiology and laboratory support**	
C-reactive protein test	26 (93)
*Clostridioides difficile* testing	23 (82)
Automated systems for identification of microorganisms	21 (75)
Molecular diagnosis for respiratory viruses	17 (61)
Galactomannan assay	12 (43)
Beta-d-Glucan testing	4 (14)
Procalcitonin test	4 (14)
**Rates and reporting of antibiotic use and outcomes**	
Antimicrobial resistance profiles	22 (77)
Rate of infections by multiresistant bacteria	19 (68)
Antimicrobial consumption (Defined Daily Dose)	18 (64)
Rates of adherence to ASP recommendations	12 (43)
Antimicrobial costs	10 (36)
Days of therapy	8 (29)
*Clostridioides difficile* infection rate	4 (14)
Reports of outcomes to administration and prescribers	11 (39)
Reports of outcomes only to hospital administration	17 (61)

ID: infectious diseases; IV: intravenously; ASP: antimicrobial stewardship program.
